# Supplementary figures and images for: Persisting symptoms three to eight months after non-hospitalized COVID-19, a prospective cohort study
Source: PLoS One. 2021 Aug 26;16(8):e0256142. doi: 10.1371/journal.pone.0256142 (PMC8389372; doi:10.1371/journal.pone.0256142)

## Flow diagram

Inclusion of participants in the Norwegian Corona Cohort

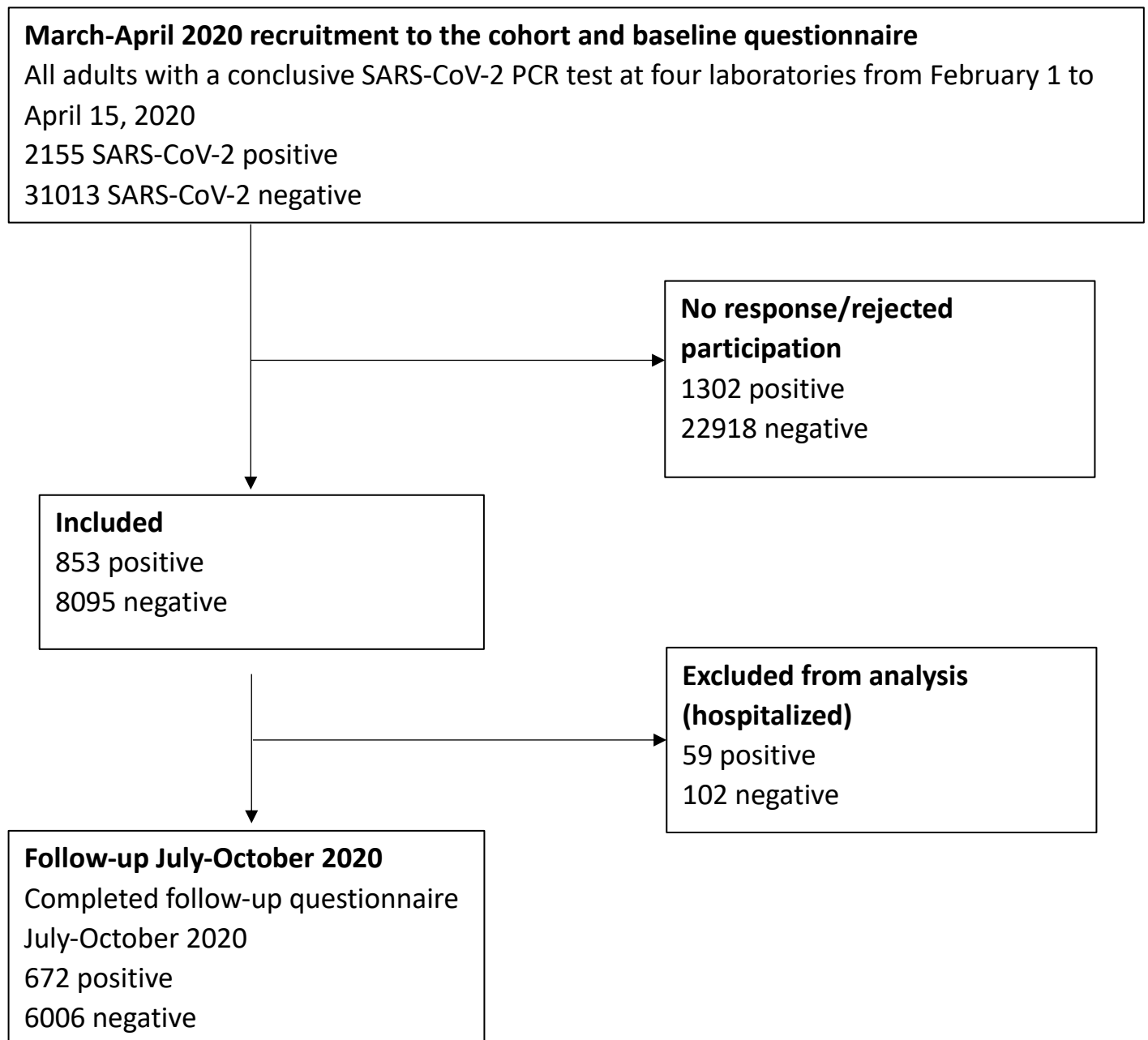

Supplement: S1 File — Flow diagram of the inclusion of participants in the Norwegian Corona Cohort. (PDF) [file pone.0256142.s001.pdf]
