# Supplementary material for: Persisting symptoms three to eight months after non-hospitalized COVID-19, a prospective cohort study
Source: PLoS One. 2021 Aug 26;16(8):e0256142. doi: 10.1371/journal.pone.0256142 (PMC8389372; doi:10.1371/journal.pone.0256142)
Supplement: S2 File — English translation of relevant parts of the questionnaires used in the study. (PDF) [file pone.0256142.s002.pdf]

Certified translation of relevant parts of the baseline questionnaire

The questionnaire was not validated as a whole, but contains items from validated questionnaires.

Check off every symptom that you have had over the course of the last three weeks:
Multiple answers are possible.

Fever
High fever (over 39)
Shortness of breath
Cough
Tiredness/exhaustion
Muscle pains Sore throat
Impaired sense of smell/taste
Blocked or runny nose
Headache
Stomach pains/nausea/diarrhoea
Other symptoms
No symptoms

Are you currently hospitalized?

Yes, due to coronavirus
Yes, but due to an illness/condition other than coronavirus
No
What illness/condition are you in hospital with?

I have previously been hospitalized due to coronavirus.

Yes
No
Don’t know

Enter your age:……..

Sex

Male
Female
Other

Are you a smoker?

Yes
No, I have never smoked
Yes, I was a smoker before
Yes, I vape
Don’t know

Check off any illnesses or conditions you have.
You can tick one option per condition.

Chronic heart disease including congenital heart disease (not high blood pressure)

Yes
No
Don’t know

High blood pressure

Yes
No
Don’t know

Chronic lung disease (other than asthma)

Yes
No
Don’t know

Asthma

Yes
No
Don’t know

Diabetes

Yes
No
Don’t know

On immunosuppressive treatment

Yes
No
Don’t know

Cancer (being treated)

Yes

No
Don’t know

I would say my physical condition is:
Poor
Average
Good

Translation of the relevant part of the follow-up questionnaire (July-October 2020)

Check off every symptom that you have had over the course of the last three weeks: Multiple answers are possible.

Fever
High fever (over 39)
Shortness of breath
Cough
Tiredness/exhaustion
Muscle pains Sore throat
Impaired sense of smell/taste
Blocked or runny nose
Headache
Stomach pains/nausea/diarrhoea
Other symptoms
No symptoms

Compared to one year ago, how would you rate your health in general now?

- Much better now than one year ago
- Somewhat better now than one year ago
- About the same
- Somewhat worse now than one year ago
- Much worse now than one year ago

This question was dichotomized in the logistic regression models and “A bit worse” and “A lot worse” were grouped together.
