# Supplementary material for: Persisting symptoms three to eight months after non-hospitalized COVID-19, a prospective cohort study
Source: PLoS One. 2021 Aug 26;16(8):e0256142. doi: 10.1371/journal.pone.0256142 (PMC8389372; doi:10.1371/journal.pone.0256142)
Supplement: Attachment — Submitted filename: PLOS_Medicine_review - rebuttal.pdf [file pone.0256142.s003.pdf]

Rebuttal letter

Dear all reviewers.

Thank you for your thorough reading of our manuscript and extensive and valuable input, and positive comments on the data which we also feel contribute to understanding Post-Acute Sequelae of SARS-CoV-2 infection (PASC). The manuscript was formatted as a letter but has now been significantly rewritten and extended to accommodate your comments. We have also updated the database with some new form submissions, and all the data is now based on the updated database.

Reviewer #1:

This article presents 3-8-month patient-reported follow-up data for non-hospitalized COVID-19 patients in Norway.

Comments:

Can the authors please provide a full list explaining the criteria for being tested? Is this a random sample of the population, or are there underlying reasons for being tested that may create bias?
For instance, it is noted in the discussion that: "At follow-up, airway symptoms were more prevalent in the SARS-CoV-2 negative group. Such symptoms were a prerequisite for being tested, and individuals frequently experiencing such symptoms are therefore expected to be overrepresented in the negative group."
A supplement has been written, which includes a description of sampling criteria. Also, the methods section has been expanded to include details about sampling. In brief, all adults tested from the onset of the pandemic to April 15 at four major laboratories in Norway were invited to participate. Testing was limited in Norway at the onset of the pandemic but was completely free. Healthcare personnel was prioritized, as were patients with risk factors for serious disease and persons with serious disease. Thus, few asymptomatic individuals were sampled as part of contact tracing efforts.

"To determine whether differences remained after adjustment for confounding, statistically significant differences in proportions were run through multivariate logistic regression models that included age, gender, income level, fitness, and smoking at baseline".
Did the authors consider including BMI and ethnicity as covariates as well?
Due to privacy concerns, BMI is omitted from the analysis dataset but can be included if it is very important. Based on reading names, we expect a very large majority of participants (>95%) to be Caucasian. This is unfortunate.

"A follow-up questionnaire was completed by 84% of the SARS-CoV-2 positive- and 74% of SARS-CoV2 negative participants between July 1 and October 30 after up to five electronic reminders. The average follow-up time was 132 days (SD=35 days) after the SARS-CoV-2 test."
The follow-up questionnaire received reasonable response rates. Can the authors please discuss whether they believe the non-responders can be assumed to be missing at random, and why?
In the discussion, we mention that knowledge of COVID-status could lead to response bias at follow-up. This section has now been expanded. It is possible that patients with particularly mild or severe disease are underrepresented. We have also included data on the responders at follow-up (age and gender) in Table 1.

We found few other differences between the groups:

Variable		TellingF2 ,00 Count	,00 Layer Column N %	1,00 Count	1,00 Layer Column N %
Age group	18-29	459	21,8%	718	10,7%
	30-39	602	28,6%	1513	22,6%
	40-49	494	23,5%	1716	25,7%
	50-59	341	16,2%	1542	23,1%
	60-69	154	7,3%	918	13,7%
	70 and older	54	2,6%	275	4,1%
Health professional	No	732	35,4%	2578	39,1%
	Yes	1336	64,6%	4011	60,9%
Gender	Female	1478	70,5%	4902	73,7%
	Male	618	29,5%	1748	26,3%
Chronic disease at baseline; cardiac, hypertension, COPD, asthma, cancer, on immuosuppresive drug and or diabets	,00	1292	61,4%	4099	61,3%
	1,00	812	38,6%	2583	38,7%
Ever smoker	,00	1191	57,5%	3741	56,6%
	1,00	882	42,5%	2865	43,4%
Fitness	Very fit	763	36,3%	2415	36,2%
	Fairly fit	1151	54,8%	3718	55,7%
	In bad shape	187	8,9%	541	8,1%

The right column is the individuals who participated at follow-up.

"Other symptoms reported more frequently at follow-up by formerly SARS-CoV-2 positive than negative participants were changes in senses of smell and taste (14% vs. 3%), fatigue (23% vs. 20%), and "other symptoms" (8% vs. 2%)."
Can the authors please provide the associated p-values alongside each stated study outcome?
The statistical testing completed is shown in the figures and tables, but the analytical techniques and tests used need to be described in the methods section in greater detail, and confidence intervals and p-values should always be quoted alongside findings in the results, conclusions and summary.
The statistics section has been updated, and p-values have been quoted alongside results.

The authors suitably note the potential for self-reporting and recall bias in the discussion section.

Overall, this is a simple analysis presenting some interesting findings. However, much more can be accomplished using this valuable and important data. There is also a lot of repetition in the text, that needs remedying.
Thank you! The text has now received a major update, and we have extended analyses on the data. The abstract has been completely rewritten to avoid repetition. Importantly, we have also found a population normal value from Norway for our outcome measure.

REVIEWER 2

This manuscript describes long-term outcomes of non-hospitalized COVID-19 patients and those who tested negative, 3-8 months after testing. While long-term sequelae of COVID-19 is an important and not well understood topic, this paper needs major revision and has several serious limitations. The abstract and the manuscript itself are almost identical. Overall, I would suggest providing much more detail in the manuscript about why this study is novel and what it contributes to the literature, the methods used for enrollment and statistical analysis, and a more complete review of the literature in the discussion. Specific recommendations are below:

Abstract:

I don't think it's appropriate to state that "Most Covid-19 patients experience relatively benign symptoms" when over 2 million people have died and over 32 million people in the US alone have been hospitalized from COVID-19. Please rephrase or specify that you are referring to outpatients only.

This has been rephrased.

For the following sentence, please state what you are comparing. You say "To determine whether differences remained," but between which groups? Please provide the exposure and outcome of interest, and then state which confounders you adjusted for and the statistical method you used.

The Methods section has been expanded and changed to account for this input.

In the results section of the abstract, please provide 95% confidence intervals for each percentage. There doesn't seem to be much a difference in fatigue between the two groups, yet you state that SARS-CoV-2 positive patients had more fatigue. I would suggest deleting that symptom or at least provide confidence intervals.

The abstract has been rewritten, and 95% confidence intervals of the mean have been included.

For this sentence, add in the percentage, "Of note, in open-ended questions, cognitive difficulties, including memory loss or concentration problems, symptoms were almost exclusively reported by the SARSCoV-2 positive patients **(XX%).** Airway symptoms were more frequently reported by SARS-CoV-2 negative participants **(XX%)**."
This part has been rewritten, and this sentence has been removed. The source of this information was open text fields in the questionnaire, but as these were partly dependent on SARS-CoV-2 status, the results are not directly comparable between groups.

How did you use logistic regression if your outcome variable is not binary? Did you dichotomize? If so please state how you did this.
The outcome was dichotomized. This has now been stated and described in the text.

Introduction:

Again, I don't think it's appropriate to state that "Most Covid-19 patients experience relatively benign symptoms" when over 2 million people have died and over 32 million people in the US alone have been hospitalized from COVID-19. Please rephrase or specify that you are referring to outpatients only.

This has been changed.

I would also suggest expanding the introduction to present what previous studies have found related to prolonged disease and complications and then state what your study adds to the literature that is novel.

Methods:

How were participants enrolled? Was it voluntary?
Yes. This is a cohort with full electronic consent from all participants. This has now been described in detail in the methods section. An Ethics section has been added.

Please provide more detail on the questionnaire: what questions were asked? Or provide the questionnaire in supplemental material.
A full translation of the baseline questionnaire has been included as supporting information.

Again, for the following sentence, please state what you are comparing. You say "To determine whether differences remained," but between which groups? Please provide the exposure and outcome of interest, and then state which confounders you adjusted for and the statistical method you used.

This has been clarified.

What do you mean by "fitness"? Is this a binary variable? Please provide details and state the definition in the methods and as a footnote in the tables/figures.

The fitness variable is a three-level variable and is presented in full in table 2 and in the supporting information.

Please list what the "other symptoms" are somewhere. Suggest adding a footnote for it in the figures and table.

Both questionnaires had items on symptoms experienced by the participants the past three weeks before completing the questionnaire. One of the items was "other symptoms," which is not further specified. However, participants could complete an open text field if they marked the "other symptoms" box, and several hundred participants did this.

Results:

Please provide 95% Cis and percentages for all results, even if you reference figures.

Confidence intervals have now been provided throughout the manuscript.

According to the figure there are symptoms that were more frequent among those who tested negative but you don't mention that in your results.

The key result of the paper is that COVID-19 patients experienced a worsening of their health three months after disease compared to one year ago. For brevity, airway symptoms that were more frequently reported by controls were only included in the figure. However, this was included in the discussion. A full table of symptoms reported at follow-up, including 95% CI has now been included in the paper and the figure has been removed. These data have now also been mentioned also in the text to give a more complete picture of the symptomatology of both groups also in the text.

You briefly mention open-ended questions but do not provide any details. Please describe these questions in the methods, include them in a supplement, and state how you summarized the results. Then provide more details in the results and percentages associated with each symptom or difficulty.

This has been completely removed from the manuscript as much more information, and new statistics have been added instead. As mentioned, some open text fields were presented to the respondents dependent on their self-reported SARS-CoV-2 status, making the comparison between the groups difficult.

Discussion:

You say the results are consistent with a study that reported 40% of survivors experienced fatigue but your estimated was 23%. That is almost half of what the other study reported, so I would not say it is consistent. Please present confidence intervals for 23%.
The paper was written when no such data existed on SARS-CoV-2. Data on SARS-CoV-2 is now available, and this paragraph has been changed to accommodate this.

Selection bias should be mentioned as a limitation. It is unclear who the patients are who are testing negative and their reason for being tested in the first place because the questionnaire does not capture reason for being tested. Do you have any data on who declined to participate in terms of demographics?
Data about the invited population has now been presented in Table 1. All adults tested in four Norwegian laboratories from testing began to April 15 were invited. Testing in Norway is completely free, but availability was limited particularly early in the pandemic. Very few asymptomatic individuals were tested. Healthcare personnel and groups with a high risk of complications from COVID-19 were prioritized.

Another limitation is that the questionnaire did not include underlying medical conditions. This is a known risk factor for long-term sequelae and could be a potential confounder between the positive cases and negative patients.
The questionnaire did include such data, and they have now been provided in Table 2.

I would suggest doing another literature search for long-term sequalae of COVID-19 and adding this to your discussion.
This has been done.

Reviewer #3

The authors report interesting results that demonstrate, in a large sample size and with a control group (patients with a negative SARS COV 2 RT PCR at baseline), that a significant proportion of patients complains of reduced quality of life and persistent symptoms after median follow up of 4 months post COVID.

Although the results deserve to be published rapidly, the format of the article needs to be improved significantly.
The current presentation does not give any adding value to the text as compared to the abstract. It seems that a letter would be sufficient unless the authors detail correctly the method used and their results
Thank you for these kind words. PLOS does not have a letter format. The manuscript has been expanded significantly. .

1- Background
A abundant literature has been recently published and we have now estimates of the frequency of symptoms in non-hospitalized patients with previous COVID-19. We also have several descriptions of the clinical profile of the symptoms. The authors should refer and discuss these articles either in the background or in the discussion section. They should compare their results to those obtained on non-hospitalized patients and not to those showing the persistence of symptoms in previously hospitalized patients which is another question.
We fully agree that non-hospitalized patients should be compared with each other and we have now conducted a new literature search.

2- Methods
The method section is too short.
This section has been expanded.
A summary of the main questions asked in the questionnaires should be provided as well as the five item RAND-36 questions (with adequate reference). What was the exact title of the question about self-rated health and how was it assessed?
RAND-36 question 2 was the outcome as this is a single-item health transition question: English version: "Compared to one year ago, how would you rate your health in general now?". This has now been included in the methods section.

The questionnaire included other RAND-36 questions, but these were not analyzed as the paper is about the experienced change of health that is experienced after COVID-19.
These items are intended to be used as part of the full RAND-36 multi-dimensional health-related quality of life calculation and are measuring aspects of current health-related quality of life. As mentioned in the discussion, the first wave of COVID-19 in Norway was dominated by individuals returning from skiing vacation in Austria and Italy and their relatives. This group had a very high self-reported fitness and also had fewer smokers, and fewer individuals with chronic disease, and their health-related quality of life can therefore not be directly compared to the normal population and our negative controls. In Table R1 the results from these questionnaire items are presented.

Table R1

		SARS-CoV-2 positive		SARS-CoV-2 negative				
**Variable**	**Level**	**N**	**% of positive**	**N**	**% of negative**	**P#**	**OR#**	**95% CI**
Self-assessed health rating	Poor	7	1 %	132	2 %	0	1.2	1.069-1.263
	Fairly good	46	7 %	609	10 %			
	Good	195	29 %	1739	29 %			
	Very good	266	39 %	2314	39 %			
	Excellent	161	24 %	1208	20 %			
Reduced work due to poor physical health (last 4 weeks)	No	591	87 %	5386	90 %			
	Yes	85	13 %	620	10 %	0.07	1.2	0.981-1.592
Done less than wanted due to poor physical health (last 4 weeks)	No	503	74 %	4835	81 %			
	Yes	173	26 %	1171	20 %	0	1.4	1.181-1.707
Limited in the tasks at work and other activities due to poor physical health (last 4 weeks)	No	600	89 %	5294	88 %			
	Yes	76	11 %	712	12 %	0.6	0.9	0.733-1.211
Trouble completing work/other activites due to poor physical health (last 4 weeks)	No	596	88 %	5512	92 %			
	Yes	80	12 %	494	8 %	0.002	1.5	1.165-1.925

RAND-36 items result by exposure group with univariate p-values and odds ratio with 95% confidence intervals.
The self-assessed health rating was better among SARS-CoV-2 positive individuals, but they reported more trouble with completing work and other activities.
The norm value in the Norwegian population for self-assessed health rating is:
Excellent: 12.4%
Very good: 36.9%
Good: 33.1%
Fairly Good: 14%
Poor: 3.6

The univariate analysis should be presented in the method and the results The respondents and non respondents should be compared in order to verify the absence of major differences between the two groups in terms of age, sex, multiplicity of symptoms. A possible bias could be a higher participation of those with initial pronounced symptoms
The methods section has been expanded as requested. It is likely that response bias influenced the results, and the discussion has now been expanded to discuss this.

The variables included in the multivariate analysis should be detailed.
These have now been detailed in the methods section.

Among the negative group, some patients have a probable COVID episode: for example those with a loss of smell and those with more than 3 symptoms suggestive of COVID.
Could the authors perform a sub analysis with these 3 groups of patients (documented, probable and negative). This could lead to more pronounced differences between groups
Thank you for this important contribution. We are aware that some of the negatives may have had COVID, and in a small sub-study, we conducted SARS-CoV-2 serology testing on 37 patients with dyspnoea, fever, and fatigue AND a negative SARS-CoV-2 PCR test during the first wave of COVID-19 in Norway and found 4 positive patients that were misclassified as negative.

The authors go too quickly from the univariate to the multivariate analysis. They should detail the variables put in the model. All the foot notes of the figures should rather go in the method section.
The relevant part of the footnote has been included in the methods section. Also, a new table with results from follow-up has been included in the manuscript.

3- Results

The result section should have different logical paragraphs : population (and potential differences with the non participating population), symptoms and self-rated health at the initial and the follow up questionnaires, factors associated with a worsening of self-rated health

Mean age should be replaced by median age +/SD for the eligible and the consenting population The symptoms present at the follow up should be described in table 1 p value between groups for all comparisons made should be given
~~A new table ...~~A new table has been made.
The rate of patients reporting spontaneously other symptoms especially cognitive and memory difficulties should be detailed?
Cognitive symptoms were not asked for, and the SARS-CoV-2 positive patients had available an extra open-text field to describe their symptoms. Direct comparison with the negative group is therefore not possible.
The type of airways symptoms investigated should be detailed.
A full description of all airway symptoms has been provided in Tables 2 and 3.

The multivariate analysis is not very clear for me. I understand that the main factor associated with a reduced self-rated health is having done a previous COVID but I do not understand what were the other factors are associated with reduced self-rated health? It is very difficult to understand the figure 2
This figure has been removed, and the multivariate analyses have been expanded and presented in a table.

4- Discussion
The discussion is rather poor and should be enriched by the numerous papers recently published on the subject of long COVID.
The limitation section is to be detailed
The discussion has been expanded.

The references section should be updated.
References have been updated.
