# Supplementary material for: Persisting symptoms three to eight months after non-hospitalized COVID-19, a prospective cohort study
Source: PLoS One. 2021 Aug 26;16(8):e0256142. doi: 10.1371/journal.pone.0256142 (PMC8389372; doi:10.1371/journal.pone.0256142)
Supplement: Attachment — Submitted filename: Reviewer comments_PLOS One.docx [file pone.0256142.s004.docx]

**Reviewer comments**

**Comments to the Author**

Thank you for the opportunity to review this manuscript which addresses an important aspect of the COVID-19 disease. The paper provides useful insight into persisting COVID-19 symptoms in a large cohort of the non-hospitalized patients. It is a helpful contribution to the literature and adds value to the body of knowledge.

**General comments**:

There are three broad areas of concern; the first is the background. The authors can provide a more elaborate study background/ introduction to justify the aim of the study.

The second is the lack of structure in the methods section. The authors should consider using a structured format (STROBE) with a careful definition of the measures assessed and a detailed data analysis plan that led to the results reported.

Another major issue in the paper's methodology was the absence of a follow-up RT PCR test. The test would help eliminate bias and confirm, especially in participants with negative baseline but with respiratory symptoms during follow-up, that they did not get infected within the 3-8 months between baseline and follow-up. Hence, the symptoms reported at follow-up. Similarly, in cases that had tested positive at baseline, there remains a possibility that they could have been re-infected during the interim period between baseline and follow-up; hence, the symptoms reported again. Current evidence suggests reinfection may occur in immunocompetent individuals shortly after recovery from mild COVID-19. [Lee JS, Kim SY, Kim TS, et al. Evidence of Severe Acute Respiratory Syndrome Coronavirus 2 Reinfection After Recovery from Mild Coronavirus Disease 2019. Clin Infect Dis. 2020 Nov 21:ciaa1421. doi: 10.1093/cid/ciaa1421.]

A repeat RT PCR test would have resolved these, as seen in several studies assessing persistent symptoms of COVID-19. [Carfì A, et al. Persistent Symptoms in Patients After Acute COVID-19. JAMA. 2020;324(6):603-5. AND Tenforde MW, et al. Symptom duration and risk factors for delayed return to usual health among outpatients with COVID-19 in a multistate health care systems network—United States, March–June 2020. Morbidity and Mortality Weekly Report. 2020;69(30):993.] If the repeat RT PCR was not done for both groups at follow-up, this should be listed as a limitation in the discussion section.

The third concern is the repetitive texts seen across the result and discussion sections. The authors can elaborate on the pathophysiology of persistent COVID-19 symptoms and how it might explain the symptoms reported by participants with positive baseline tests. Also, the authors should correct the incomplete citation of references 3,5 and 12.

Specific comments are stated below:

**Introduction:**

1. The authors should provide a more elaborate background to the study aim. For example, what is the burden of COVID-19 disease in the population studied (Norway)? What does the current literature on the pathophysiology of COVID-19 suggest? What gap in literature creates a justification for the present study? A sentence or two on the potential implication of the findings from the study aim?
2. Please take special note that acronyms should be defined the first time they are used in the manuscript. For example, COVID-19, SARS-CoV-2, and RT PCR.

**Methodology:**

1. The authors should consider providing structure to the methods section, perhaps using the Strengthening the Reporting of Observational Studies in Epidemiology (STROBE) framework as a guide [Vandenbroucke JP, et al. STROBE Initiative. Strengthening the Reporting of Observational Studies in Epidemiology (STROBE): explanation and elaboration. PLoS Med. 2007 Oct 16;4(10):e297.]

For example, explicit statements on the following sections with sub-headers, in the following order: Study design and study population; Ethics approval; Measures and instruments; and Statistical analysis.

2. Page 4, line 8: The authors should define the measure “fitness” in the methodology?
3. Page 4, line 12: The authors should state the criteria used to stratify or categorize “COVID-19 severity of disease”? And define the different categories. For example, what constituted a mild or severe disease in the current study.
4. Were symptoms present even before the COVID-19 infection? This is an important information since some patients might have confounding comorbidities. The authors should specify if this information was taken into account and possibly elaborate in the Discussion section.
5. For those who reported persistent symptoms, was there any procedure to rule out any illness asides from COVID-19 disease? Or by reporting seemingly persistent COVID-19 like symptoms, was it implicitly assumed that the symptom must be linked to the former COVID-19 disease in the positive patients at baseline. If no measure was taken, this may be stated as part of the study limitations.
6. Were participants tested for SARS-CoV-2 infection again before the follow-up questionnaire was administered to confirm “persistent symptoms”? Several studies have reported cases of reinfection in immunocompetent individuals shortly after recovery from mild COVID-19. [Example: Lee JS, Kim SY, Kim TS, et al. Evidence of Severe Acute Respiratory Syndrome Coronavirus 2 Reinfection After Recovery from Mild Coronavirus Disease 2019. Clin Infect Dis. 2020 Nov 21:ciaa1421. doi: 10.1093/cid/ciaa1421.] If participants were not tested at follow-up, this should also be listed as a limitation.
7. The authors should state if therapy (medications etc.) taken by participants that were positive at baseline were considered when collecting data as it is a possible confounding factor.

**Results:**

1. The sentence “A total of 8786 participants were included.” can be rephrased to “A total of 8786 participants were enrolled in the study”.
2. Table 1: The proportion of females who were negative for SARS-CoV-2 and complete the questionnaire at 3 to 8 months was stated as 76%. The proportion should be 75% (4520/6006 X 100). Kindly correct.
3. Table 2 can be better organized with OR (95% CI) placed in a single column.
4. How were the variables “Fitness- bad shape, fairly fit, fit” AND “Household income- <300k, 300-600k, 600-1000k, and >1000k” analyzed? The authors can provide more clarity by stating the reference as done previously. For example, was the reference "bad shape" for Fitness and "<300k" for NOK?
5. Table 2: The variable Fitness has a p-value <0.001, however, the OR- 2.0 is not within the 95% CI range 0.44-0.57. Can the authors clarify?
6. Table 3: The statement “The average follow-up time was 132 days (SD=35 days) after testing (Table 3)” is not indicated in table 3.
7. Table 3 demonstrates a significant percentage of participants negative at baseline now presenting with COVID-like symptoms (cough, sore throat, body ache, nasal symptoms and headache) and a higher proportion of the negative participants presenting symptomatically compared to positive participants at baseline. The challenge with interpreting these findings is the absence of follow-up SARS-CoV-2 tests on both groups. This is because it is possible that some of the patients who had been negative at baseline over the 3-8 months of follow-up were infected and might have self-isolated and not reported for testing at the study centres. Hence, the symptoms subsequently observed during the follow-up review.

So, we cannot with all certainty infer that participants with +ve RT PCR at baseline had higher odds of “persistent symptoms” than the population assessed who were -ve at baseline assessment. Instead, we can infer that they perceived a significant change in their health status based on the measure "Self-assessed change in health last 1 year (summer)".

8. Page 11, line 3: The authors should correct the "gender (p=0,8)" to "Neither gender (p=0.8)".
9. Table 4: What was the reference value for the “Household income”? The authors should include this as part of the footnote labelled “d”
10. Page 12, line 18-22. There is some repetition of the results, specifically with the text supporting Figure 3.

**Discussion**

1. Page 13, line 11-12: The statement “where healthcare workers and patients with risk factors for severe disease were prioritized and therefore overrepresented in the negative group” should be revised. While healthcare workers were overrepresented in the negative group, the same does not apply to participants with risk factors for severe diseases. For example, in the negative group, only 39% had chronic diseases, and 61% did not; similarly, about 44% were smokers, and 56% were not.
2. The authors could include a paragraph discussing the pathophysiological approach of the subject of persistent Covid-19 symptoms, referring to the hypothesis proposed in the literature, such as inflammatory post-infectious syndrome, vasculitis, endothelial dysfunction, and social isolation. The authors can refer to the articles:

Sollini M, et al. Vasculitis changes in COVID-19 survivors with persistent symptoms: an [18F] FDG-PET/CT study. Eur J Nucl Med Mol Imaging. 2020. **OR** Libby P, Lüscher T. COVID-19 is, in the end, an endothelial disease. Eur Heart J. 2020;41:3038–44. **OR** Garg P, et al. The "post-COVID" syndrome: how deep is the damage? J Med Virol. 2020:1–2.

**References**

1. Ref 3: Correct to- Rogers JP, Chesney E, Oliver D, Pollak TA, McGuire P, Fusar-Poli P, et al. Psychiatric and neuropsychiatric presentations associated with severe coronavirus infections: a systematic review and meta-analysis with comparison to the COVID-19 pandemic. Lancet Psychiatry. 2020 Jul;7(7):611-627. doi: 10.1016/S2215-0366(20)30203-0.
2. Ref 5: Correct to- Troyer EA, Kohn JN, Hong S. Are we facing a crashing wave of neuropsychiatric sequelae of COVID-19? Neuropsychiatric symptoms and potential immunologic mechanisms. Brain Behav Immun. 2020 Jul;87:34-39. doi: 10.1016/j.bbi.2020.04.027.
3. Ref 12: Correct to- Huang C, Huang L, Wang Y, Li X, Ren L, Gu X, et al. 6-month consequences of COVID-19 in patients discharged from hospital: a cohort study. Lancet. 2021 Jan 16;397(10270):220-232. doi: 10.1016/S0140-6736(20)32656-8.
4. References 6 and 7 can be replaced with peer-reviewed articles.
